# Glycolysis gene expression profilings screen for prognostic risk signature of hepatocellular carcinoma

**DOI:** 10.18632/aging.102489

**Published:** 2019-12-02

**Authors:** Longyang Jiang, Lan Zhao, Jia Bi, Qiutong Guan, Aoshuang Qi, Qian Wei, Miao He, Minjie Wei, Lin Zhao

**Affiliations:** 1 Department of Pharmacology, School of Pharmacy, China Medical University, Shenyang North New Area, Shenyang 110122, Liaoning, China; 2 Liaoning Key Laboratory of Molecular Targeted Anti-Tumor Drug Development and Evaluation, China Medical University, Shenyang North New Area, Shenyang 110122, Liaoning, China

**Keywords:** hepatocellular carcinoma, glycolysis, signature, prognostic, risk

## Abstract

Metabolic changes are the markers of cancer and have attracted wide attention in recent years. One of the main metabolic features of tumor cells is the high level of glycolysis, even if there is oxygen. The transformation and preference of metabolic pathways is usually regulated by specific gene expression. The aim of this study is to develop a glycolysis-related risk signature as a biomarker via four common cancer types. Only hepatocellular carcinoma was shown the strong relationship with glycolysis. The mRNA sequencing and chip data of hepatocellular carcinoma, breast invasive carcinoma, renal clear cell carcinoma, colorectal adenocarcinoma were included in the study. Gene set enrichment analysis was performed, profiling three glycolysis-related gene sets, it revealed genes associated with the biological process. Univariate and multivariate Cox proportional regression models were used to screen out prognostic-related gene signature. We identified six mRNAs (DPYSL4, HOMER1, ABCB6, CENPA, CDK1, STMN1) significantly associated with overall survival in the Cox proportional regression model for hepatocellular carcinoma. Based on this gene signature, we were able to divide patients into high-risk and low-risk subgroups. Multivariate Cox regression analysis showed that prognostic power of this six gene signature is independent of clinical variables. Further, we validated this data in our own 55 paired hepatocellular carcinoma and adjacent tissues. The results showed that these proteins were highly expressed in hepatocellular carcinoma tissues compared with adjacent tissue. The survival time of high-risk group was significantly shorter than that of low-risk group, indicating that high-risk group had poor prognosis. We calculated the correlation coefficients between six proteins and found that these six proteins were independent of each other. In conclusions, we developed a glycolysis-related gene signature that could predict survival in hepatocellular carcinoma patients. Our findings provide novel insight to the mechanisms of glycolysis and it is useful for identifying patients with hepatocellular carcinoma with poor prognoses.

## INTRODUCTION

Cells can utilize multiple metabolic pathways for energy production and biosynthesis depending on the requirements for cellular function and the availability of metabolites [1]. A major hurdle in neoplastic transformation is the ability of cells to meet the high bioenergetic and biosynthetic needs necessary to sustain cancer cell growth. It is well established that cancer cells shift to a pro-anabolic metabolism induced by oncogenes, such as c-Myc [2]. Most notable is the Warburg effect wherein cancer cells increase glycolysis and it is a metabolic hallmark of virtually all cancer cells, characterized by excessive conversion of glucose to lactate. It occurs in most organisms, even though in the presence of oxygen. An increase in glycolysis provides cancer cells with energy and heightened potential for biomass production from glycolytic intermediates [3]. Glycolysis was an early attractive target for cancer therapy given the clinical observation that many tumors exhibit a significant increase in glucose uptake compared with adjacent normal tissue [4]. LDH-A, a will pyruvate (end product of glycolysis) converted to lactic acid metabolic enzymes, is identified as the first metabolic target of the oncogene MYC [5]. Another potential therapeutic target is the glycolytic protein HK2. Many tumor cells overexpress HK2, and preclinical mouse models of genetically engineered NSCLC (non-small cell lung cancer) and breast cancer demonstrate that HK2 inhibition delays tumor progression [6]. Uncontrolled growth, reduced ability to undergo apoptosis and the ability to metastasize are some of the important features of malignancies, regardless of origins of tissues [7]. Therefore, early detection of cancer is always one of the most effective ways to improve the overall survival rate of cancer patients. Clinically, better or alternative methods to identify cancer risk groups are always needed [8]. Thousands of biomarkers that may be associated with survival and prognosis have been explored through various methods. However, we found that limited studies have systematically investigated the metabolic status and its prognostic value in patients with tumor. And most biomarkers have been developed in a wide range, not selected for specific features or single signaling pathways, which could be a new entry point for us to research. Therefore, clarifying the relationship between glycolysis and tumor is crucial for understanding the mechanism of tumorigenesis.

In the present study, we performed GSEA to identify glucose-related gene sets that could distinguish the clinical and molecular variables of several common cancers which were mentioned above, including colon adenocarcinoma (COAD), kidney renal clear cell carcinoma (KIRC), hepatocellular carcinoma (HCC) and breast invasive carcinoma (BRCA). And then, we found the significant mRNAs could be mined only in HCC. We developed a glucose-related prognostic signature with the whole genome expression data from the TCGA database for the patients with HCC in the TCGA dataset. Surprisingly, the local glycolysis-related risk signature could independently classify patients with HCC with a high risk of unfavorable outcome. We first identified a glycolysis-related risk signature for patients with HCC. These results might provide a new view for the research of HCC and individual treatment.

## RESULTS

### Glycolysis-related gene sets differ significantly between adjacent cancer samples and tumor samples

**Figure 1:**
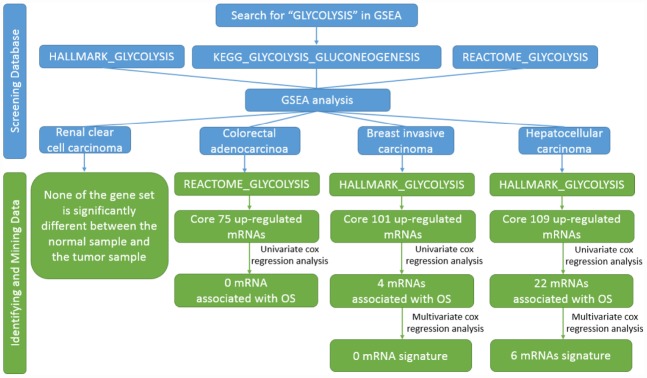
**Flow chart of finding six mRNAs signature in HCC.**

**Figure 2:**
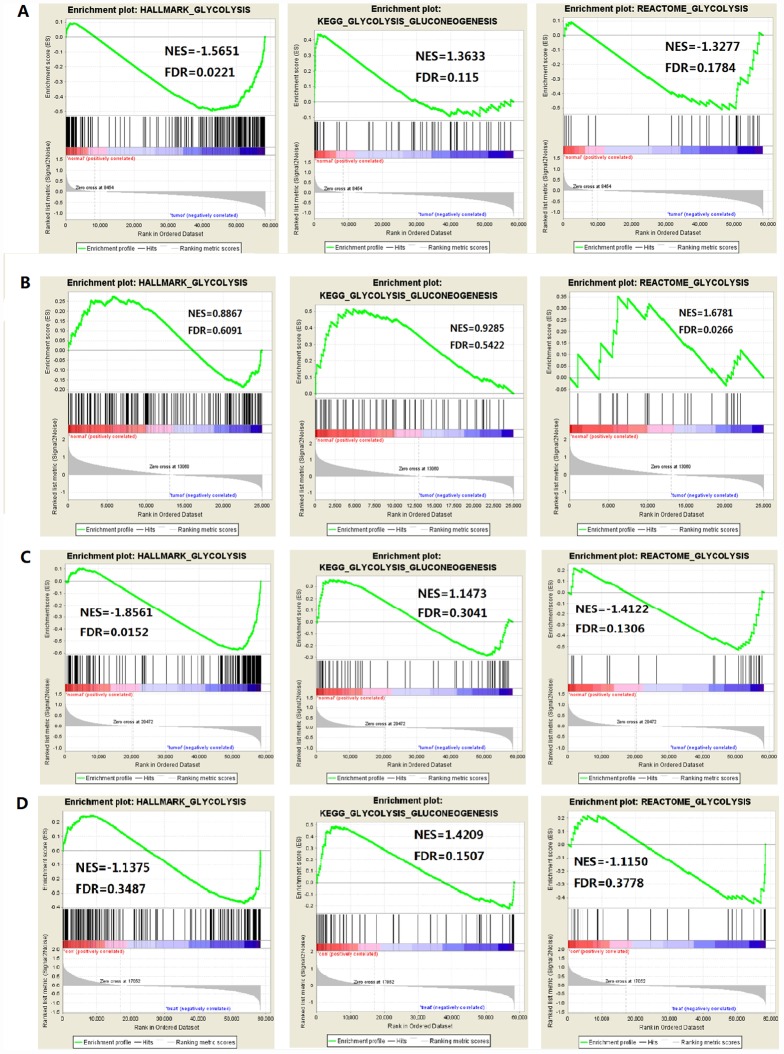
**Enrichment plots of three glycolysis-related gene sets in each tumor (FDR is the P value after correction by multiple hypothesis test).** (**A**) HCC, (**B**) Colorectal adenocarcinoma, (**C**) Breast invasive carcinoma, (**D**) Renal clear cell carcinoma.

The study included four solid tumors, including COAD, KIRC, HCC and BRCA. The mRNA expression and clinical data of all patients were obtained from the Cancer Genome Atlas Database (TCGA). We found all glycolysis-related gene sets on the Molecular Signatures Database v4.0 (http://www.broadinstitute.org/gsea/ msigdb/index.jsp), namely three different gene sets (HALLMARK_GLYCOLYSIS, KEGG_GLYCOLYSIS_ GLUCONEOGENESIS, REACTOME_GLYCOLYSIS). First, we used GSEA to explore whether these three glycolysis-related gene sets differ significantly between adjacent cancer samples and tumor samples (Figure 1). We found that the HALLMARK_GLYCOLYSIS gene set in HCC was significantly different between paracancerous and tumor samples (FDR=0.0221); the REACTOME_GLYCOLYSIS gene set in COAD was significantly different between paracancerous and tumor samples (FDR=0.0266). The HALLMARK_ GLYCOLYSIS gene set in BRCA was significantly different between the paracancerous sample and the tumor sample (FDR = 0.0152). In the analysis of renal clear cell carcinoma, the FDR values of three gene sets were all found to be greater than 0.05 (Figure 2), indicating that there is no significant difference between the adjacent glycolysis-related gene sets in the paracancerous samples and the tumor samples. Next, the core genes (CORE ENRICHMENT: YES) under the above gene set is screened, that is, the gene whose expression is up-regulated in the tumor tissue. Among the four solid tumors, differentially expressed genes can be screened for COAD, HCC and BRCA. Compared with adjacent tissues, 75 up-regulated mRNAs were screened in COAD tissues; 109 up-regulated mRNAs were screened in HCC tissues compared with adjacent tissues; compared with adjacent tissues, a total of 101 up-regulated mRNAs were screened in BRCA tissues (Table 1).

**Table 1:** Core genes of three cancers.

**Cancer**	**Gene**	**CORE ENRICHMENT**
HCC	SLC25A10, CHST6, STC2, EGLN3, MET, GLCE, PRPS1, HK2, PPFIA4, PAM, MIOX, DPYSL4, PHKA2, HDLBP, GALK1, B3GNT3, VCAN, DDIT4, ZNF292, COL5A1, TGFBI, NDUFV3, HOMER1, GALK2, EXT2, CHST1, VEGFA, SLC16A3, HS2ST1, GFPT1, ME2, B4GALT4, GAL3ST1, STC1, MDH1, SDC2, ME1, BIK, TPI1, MPI, GALE, SOX9, AGRN, ARTN, PGK1, COPB2, CHST12, SDHC, ARPP19, G6PD, ENO1, RPE, SRD5A3, ABCB6, PLOD1, FKBP4, NSDHL, GYS1, KDELR3, NANP, MDH2, SPAG4, PGLS, MIF, TALDO1, B4GALT2, KIF2A, ALDOA, TSTA3, SAP30, TXN, CHPF2, NASP, B3GALT6, RRAGD, PYGB, HSPA5, GNPDA1, NOL3, ECD, DEPDC1, EFNA3, PAXIP1, POLR3K, RARS, BPNT1, CENPA, GPC3, B3GAT3, GMPPB, ALG1, KIF20A, CDK1, RBCK1, GMPPA, STMN1, HAX1, MED24, HMMR, XYLT2, AURKA, IDUA, CLN6, PSMC4, PPIA, ANKZF1, COG2, B4GALT7, P4HA2	YES
COAD	PPP2CB, PFKL, PFKFB2, PGAM1P5, PPP2R5D, PPP2R1A, PPP2CA, ALDOA, ADH1B, ADH1C, PGM1, ACSS2, PCK1, ADH5, GALM, ADH1A, GCK, ADH6, ALDH3A2, HK2, ALDH9A1, ALDH2, DLD, PDHB, PCK2, HK1, DLAT, CITED2, UGP2, CAPN5, MXI1, FAM162A, DSC2, DCN, MPI, GOT1, ME2, BPNT1, SLC35A3, PC, AK3, EXT1, MDH1, TGFA, PKP2, AGL, LHPP, VLDLR, B4GALT1, GNE, EGFR, B4GALT4, ISG20, PLOD2, GMPPB, SDHC, CHST2, ADORA2B, EGLN3, CYB5A, IL13RA1, CASP6, GYS1, COG2, CACNA1H, ANG, IDH1, NDUFV3, B3GNT3, PGAM1, CLDN3, CSDC2, ELF3, CTH, GAL3ST1	YES
BRCA	P4HA2, CACNA1H, ARTN, PGK1, AURKA, BIK, CHPF, FAM162A, SDC1, TSTA3, CXCR4, FUT8, ELF3, GOT2, NASP, P4HA1, GALE, SRD5A3, EFNA3, PLOD1, PGLS, SLC16A3, GFPT1, CLDN3, PDK3, SLC25A13, PMM2, TFF3, PRPS1, GALK1, B4GALT7, SLC37A4, SLC25A10, VEGFA, TPI1, MED24, FKBP4, SPAG4, HAX1, SDHC, PSMC4, GMPPB, LDHA, XYLT2, SLC35A3, CDK1, MDH2, HSPA5, TPBG, SOD1, PGM2, ALG1, B3GAT3, MIF, CHST6, PPIA, ALDOA, B4GALT4, CASP6, GPC1, AGRN, TXN, PAXIP1, IDUA, B3GALT6, CLN6, GNPDA1, VCAN, ISG20, MIOX, B4GALT2, HDLBP, DEPDC1, RPE, KDELR3, COG2, HMMR, PGAM1, STMN1, KIF20A, EGLN3, RBCK1, ENO2, COL5A1, POLR3K, GPC4, B4GALT1, PFKP, SAP30, RARS, GMPPA, ME2, QSOX1, NSDHL, TALDO1, CENPA, COPB2, BPNT1, IER3, AKR1A1, CHPF2	YES

**Figure 3:**
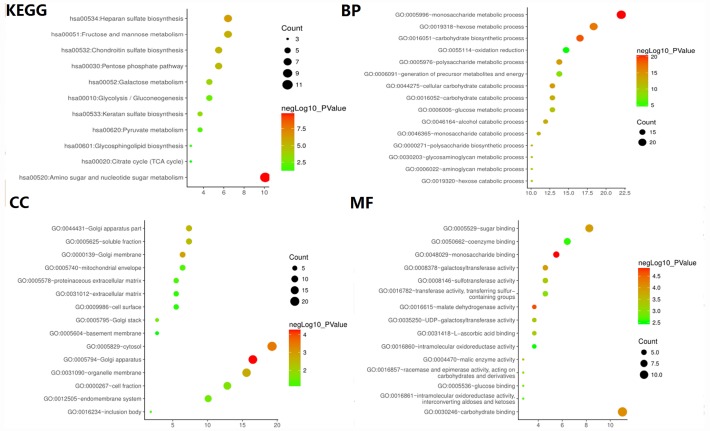
**GO and KEGG pathway enrichment analysis of glycolysis-related genes selected from GSEA.**

To verify whether the core gene is involved in glycolysis, we conducted an in-depth study using GO analysis and KEGG pathway enrichment analysis. The results showed that the most enriched biological process term (BP) was related to glucose metabolism process, molecular function term (MF) was related to sugar binding and glucose binding, and KEGG pathway enrichment analysis involved glycolysis/gluconeogenesis, suggesting that the selected core genes are indeed related to glycolysis (Figure 3).

### Identification of glycolysis-related genes associated with patients’ survival

**Figure 4:**
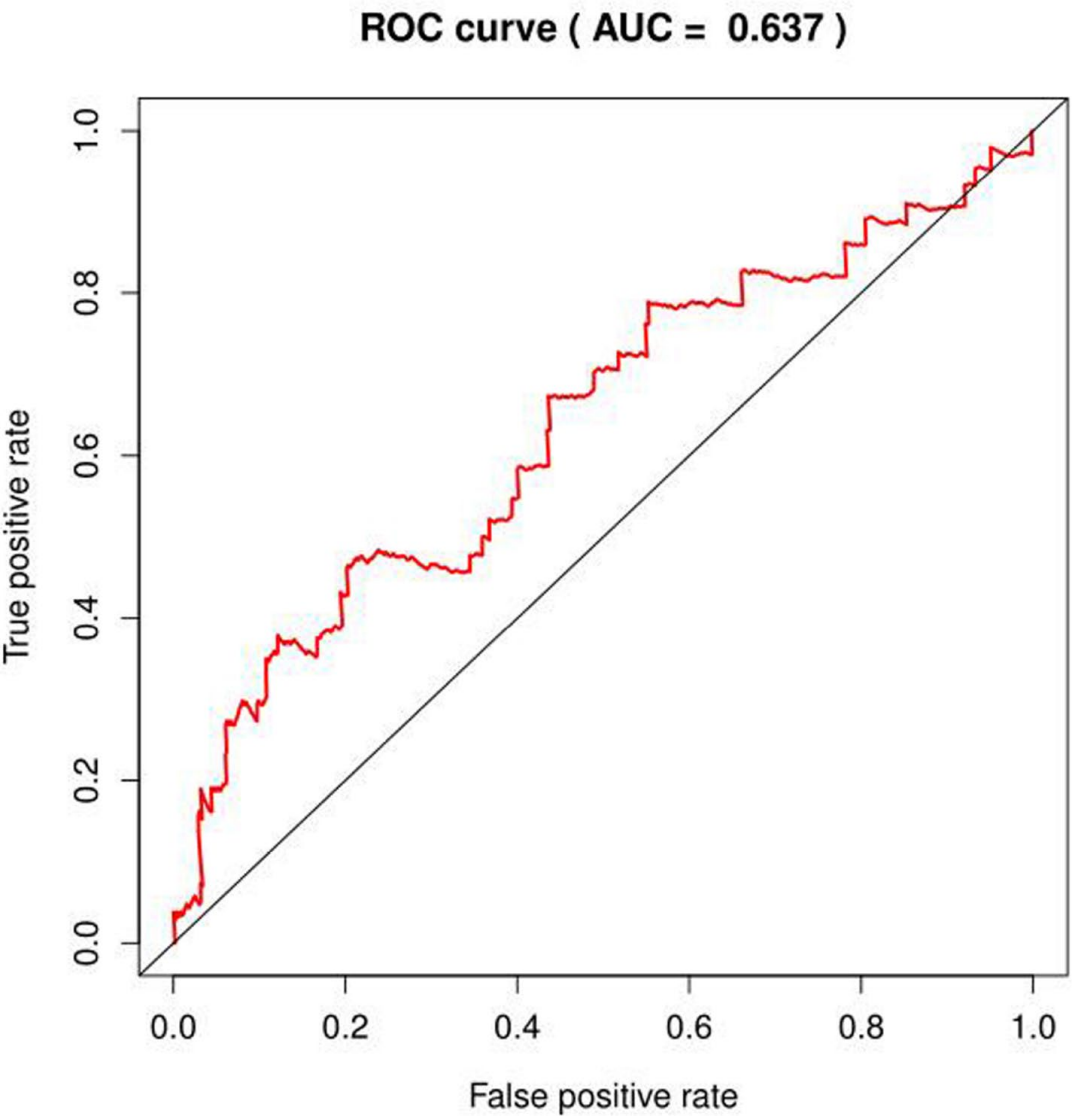
**ROC curve of glycolysis-related genes in BRCA.**

We selected the core genes from the GSEA results table and performed a univariate Cox regression analysis to further screen for the mRNAs associated with the patient's OS. It was found that 22 mRNAs were selected for HCC in patients with OS (*P*<0.001); in the analysis of COAD, no mRNA associated with OS was found; in the analysis of BRCA, four mRNAs were associated with OS (*P* < 0.1, Table 2), but the area under the curve (AUC = 0.637) of the receiver operating characteristic curve (ROC), indicating diagnostic performance was less than 0.70 (Figure 4). The efficacy is not high, therefore, no prognostic genes related to glycolysis can be screened in breast invasive cancer. The univariate Cox proportional regression model found that the differentially expressed genes related to glycolysis which were associated with the patient's OS could only been selected in HCC of the remaining three tumors.

**Table 2:** The result of univariate Cox analysis in BRCA.

**mRNA**	**HR**	**z**	***p* value**
P4HA2	1.455667226	2.329155281	0.019850841
CACNA1H	1.122726446	2.207138132	0.02730441
ARTN	1.162671204	2.179141916	0.029321127
PGK1	1.361430124	2.074462111	0.038036414

Multivariate Cox regression analysis further examined the relationship between glycolysis gene expression profiles and OS in patients with HCC. Six mRNAs (DPYSL4, HOMER1, ABCB6, CENPA, CDK1, STMN1) were screened as independent prognostic indicators (Table 3). As gene signature, they could be classified into dangerous (DPYSL4, HOMER1, ABCB6, CENPA, STMN1, HR> 1) and protected type (CDK1, HR<1).

**Table 3:** Details of the six selected mRNAs.

**mRNA**	**Ensemble ID**	**Chromosome location**	**β(Cox)**	**HR**	***p***
DPYSL4	ENSG0000015164	Chr10:132184983..132205776	0.1142	1.1210	0.000816
HOMER1	ENSG00000152413	Chr5:79,372,636-79,514,217	0.1982	1.2192	0.000349
ABCB6	ENSG00000115657	Chr2:219,209,766-219,219,017	0.2647	1.3030	0.000461
CENPA	ENSG00000115163	Chr2:26,764,289-26,801,067	0.4603	1.5846	8.66E-06
CDK1	ENSG00000170312	Chr10:60,778,331-60,794,852	-0.5359	0.5852	6.04E-05
STMN1	ENSG00000117632	Chr1:25,884,181-25,906,991	0.3966	1.4867	1.56E-06

**Figure 5:**
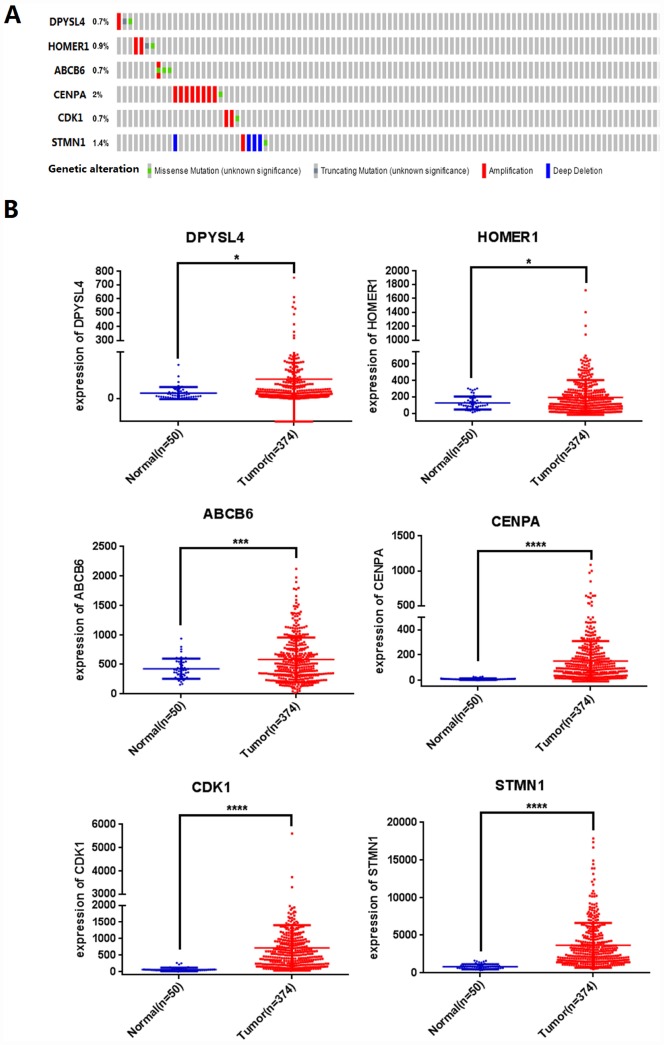
**Identification of prognostic risk signature associated with glycolysis.** (**A**) Mutations of selected genes in patients with hepatocellular carcinoma, (**B**) Differential expression analysis of 6 selected genes. (*p<0.05, ***p<0.001, ****p<0.0001).

Next, we analyzed the mutations of the selected six mRNAs in HCC through clinical HCC samples in the cBioPortal online web database. The results showed that 19 of 309 patients (6%) had mutations. Among them, DPYSL4: one case of gene amplification, one case of missense mutation, one case of truncation mutation; 0.9% of HOMER1 gene mutation, two cases of amplification, one case of missense mutation, one case of truncation mutation; 0.7% of ABCB6 gene mutation; CENPA gene has 2% mutation; CDK1 gene mutation is 0.7%; STMN1 gene mutation accounts for 1.4% (Figure 5A).

We further analyzed the differential expression of DPYSL4, HOMER1, ABCB6, CENPA, CDK1, STMN1 in cancer and adjacent tissues, and found that compared with adjacent tissues, the six genes in 309 HCC tissues were significantly up-regulated (*P*<0.01, Figure 5B).

### Construction and validation of a six-mRNA signature for predicting patients’ outcome

**Figure 6:**
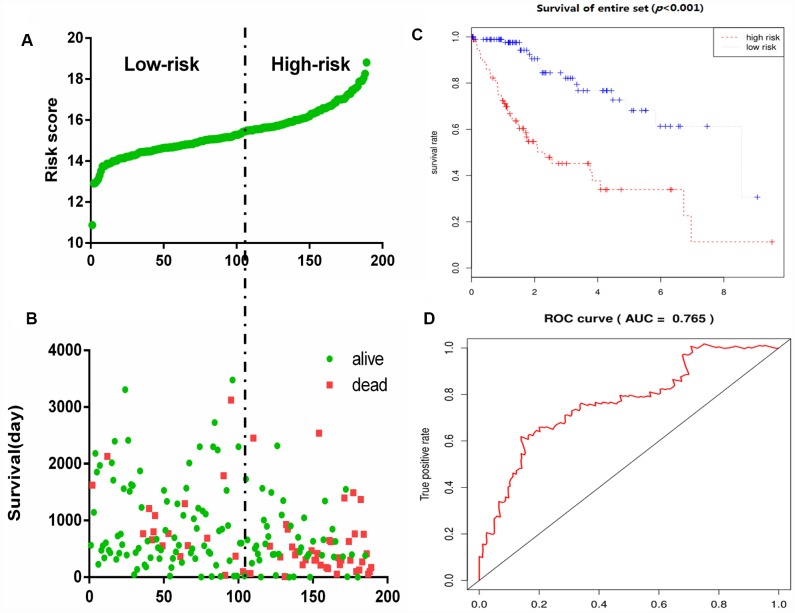
**Glycolysis-related gene signature predicts OS in patients with HCC.** (**A**) Distribution of risk scores per patient, (**B**) Relationship between survival days and survival status of each patients, (**C**) K-M curve to verify the predictive effect of gene signature, (**D**) ROC curve analysis to evaluate the diagnostic efficacy of gene signature.

To construct a prognostic signature, these six mRNAs were analyzed using a multivariate Cox regression analysis in the entire dataset with survival. Next, by integrating the expression profiles of six mRNAs and the corresponding regression coefficients obtained from the above multivariate Cox regression analysis, a prognostic feature was constructed, as shown below: Risk score = (0.1142 × expression level of DPYSL4) + (0.1982 × expression level of HOMER1) + (0.2647 × expression level of ABCB6) + (0.4603 × expression level of CENPA) + (-0.5359 × expression level of CDK1) + (0.3966 × expression level of STMN1). Using six mRNA signatures, we calculated the risk scores for each patient in the entire data set and ranked them in order of increasing risk scores (Figure 6A). Thus, 309 patients in the entire data set were classified as high risk groups (n = 154) and low-risk groups (n = 155), using the median risk score as the threshold. The Kaplan-Meier (KM) analysis showed a significant difference in the outcome of the patients between the high-risk group and the low-risk group (log-rank test *P* <0.001; Figure 6C). The high-risk subgroup had significantly worse survival than those in the low risk subgroup. To evaluate how well the six-mRNA signature for diagnosis, the ROC curve analysis was carried out. The AUC for the six-mRNA signature was 0.765 (Figure 6D), demonstrating the good diagnostic significance of six-mRNA signature for survival prediction in the entire dataset. Figure 6B showed the risk score, OS (in days) and life status of 309 patients in the entire data set, ranked in order of increased risk score, the patients with high-risk scores had higher mortality rates than did the patients with low-risk scores.

We applied chi-square test to reveal the relation between risk score and clinical features (Table 4). It revealed that T, N, M, stage, grade, relative family history concerned the risk score of HCC patients.

**Table 4:** The chi-square test of the relation between risk score and clinical features.

**Clinical feature**	**Risk score**		**X^2^**	***p***
	**High risk n(%)**	**Low risk n(%)**		
Gender			0.057	0.811
Male	92(44.4%)	115(55.6%)		
Female	43(43.0%)	57(57.0%)		
Age			0.318	0.573
≥61	72(48.6%)	86(54.4%)		
<61	64(42.4%)	87(57.6%))		
T			26.629	< 0.001
T1	46(30.5%)	105(69.5%)		
T2	36(48.0%)	39(52.0%)		
T3	43(63.2%)	25(38.6%)		
T4	9(75.0%)	3(25.0%)		
N			6.203	0.013
N0	84(38.5%)	134(61.5%)		
N1	4(100%)	0(0.0%)		
M			5.623	0.018
M0	93(41.0%)	134(59.0%)		
M1	4(100%)	0(0.0%)		
Stage			39.396	<0.001
I	39(27.3%)	104(72.7%)		
II	31(44.9%)	38(55.1%)		
III	50(67.6%)	24(32.4%)		
IV	5(100%)	0(0.0%)		
Grade			14.656	0.002
I	25(56.8%)	19(43.2%)		
II	66(47.8%)	72(52.2%)		
III	44(40.0%)	66(60.0%)		
IV	0(0.0%)	13(100%)		
Person neoplasm cancer status			2.96	0.085
Tumor free	51(37.8%)	84(62.2%)		
With tumor	49(49.0%)	51(51.0%)		
New tumor event after initial treatment			1.18	0.179
No	48(39%)	75(61%)		
Yes	58(47.5%)	64(52.5%)		
Relative family history			5.868	0.015
No	65(35.9%)	116(64.1%)		
Yes	48(51.15)	46(48.9%)		
Adjacent hepatic tissue inflammation extent type			3.619	0.164
None	47(52.2%)	43(47.8%)		
Mild	34(39.1%)	53(60.9%)		
Severe	3(33.3%)	6(66.7%)		

Abbreviations:T: Tumor; N: Node (regional lymph node); M: Metastasis

**Figure 7:**
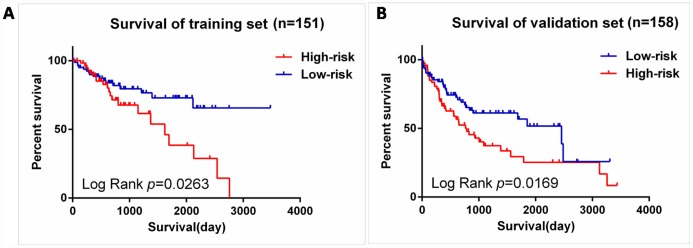
**Validation for prognostic value of risk signature.** (**A**) K-M curves for train set, (**B**) K-M curves for validation set.

We next validated our six-mRNA signature in the training set and validation set to confirm our findings. 309 patients were randomly divided into a training set (n = 151) and a validation set (n = 158). The ID of the two groups please see the Supplementary Table 1. Cross validation showed that the CLF score was 0.82, indicating that the grouping was stable and reliable. Consistent with the results of the entire set, patients in the high-risk group had significantly shorter survival time than those in the low-risk group of the training set (log-rank test *P* =0.0263; Figure 7A). The validation data set, similar results were observed: the survival rate of patients in the high-risk group was significantly lower than that in the low-risk group (log-rank test *P* =0.0169; Figure 7B).

**Figure 8:**
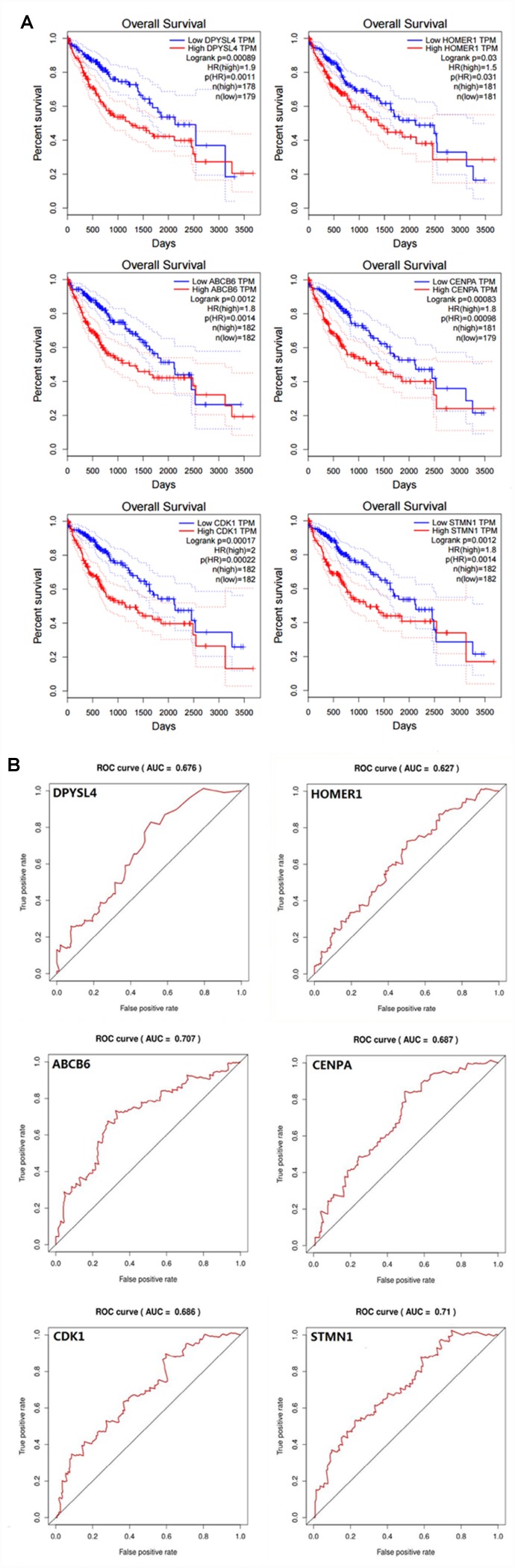
**Verifying the prognostic and diagnostic value of risk signature is better than single biomarker.** (**A**) K-M analysis, (**B**) ROC curve.

In order to confirm that the gene signature is more significant than the single gene biomarker, we verified it with the KM analysis and the ROC curve and the results showed that our hypothesis is correct. When the six genes act as a single biomarker respectively, their diagnostic significance was not better than the six-mRNA signature (Figure 8).

### Independence of the risk score for the six-mRNA from other clinical variables

**Figure 9:**
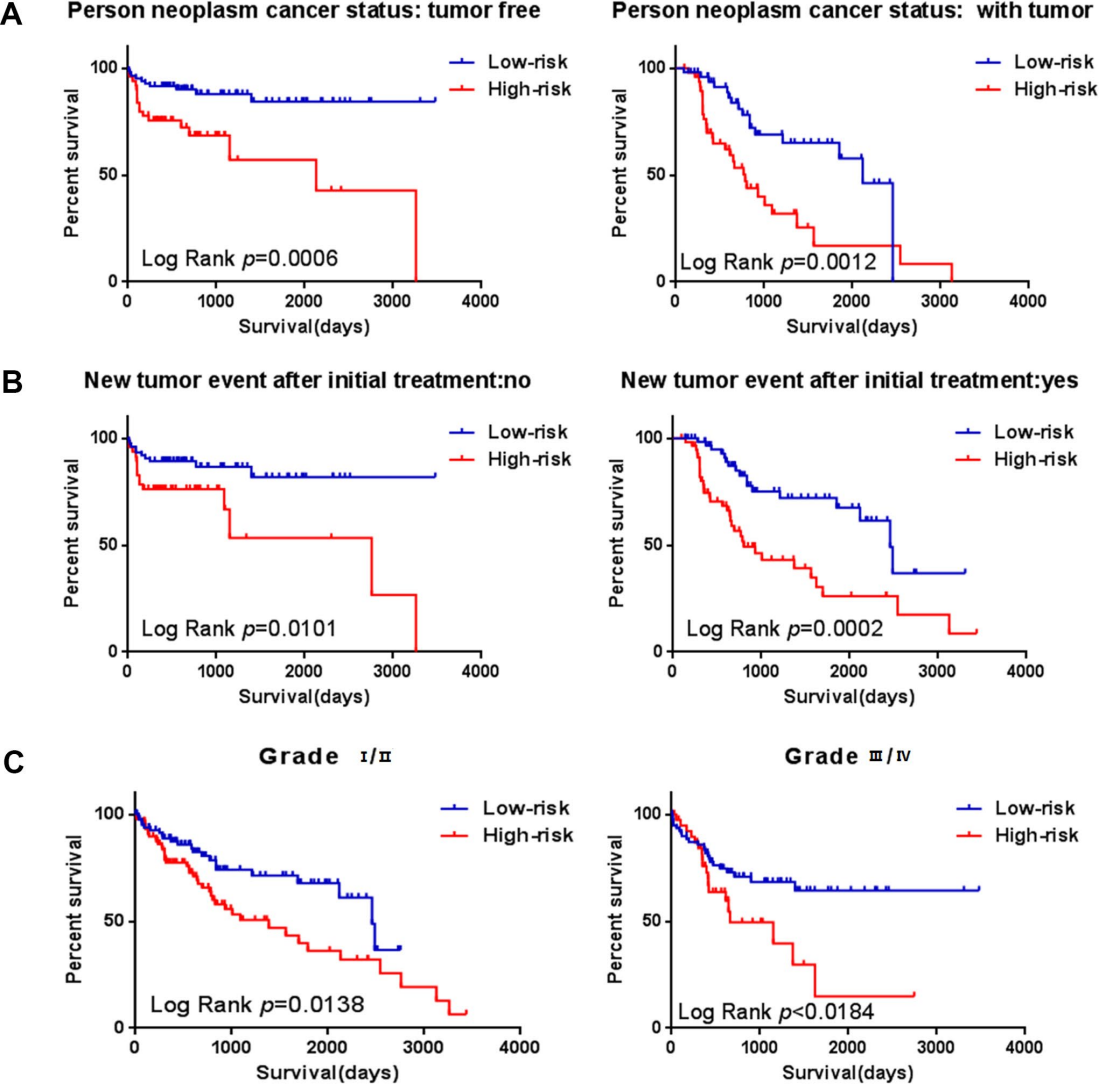
**Stratified analysis for prognostic value of risk signature for the patients.** (**A**–**B**) Stratified analysis for patients divided into person neoplasm cancer status, (**C**–**D**) Stratified analysis for patients divided into new tumor event after initial treatment, (**E**–**F**) Stratified analysis for patients divided into grade.

To assess whether the prognostic ability of the six mRNA markers is independent of other clinical parameters including family history, new tumor event after initial treatment, neoplasm cancer status and tumor stage, the univariate and multivariate Cox regression analyses were performed in the entire dataset. The results of the data showed that the risk score of six mRNA is significantly related to patients’ survival. Surprisingly, the six-mRNA signature still retained an independent prognostic indicator after adjustment for other clinical parameters in the entire dataset (*P*-value<0.001, HR = 1.884, 95% CI = 1.287-2.757; Table 5 and Table 6). In addition, we found that T, neoplasm cancer status, new tumor event after initial treatment were also independent prognostic factors, because they had significant differences not only in the univariate analysis, but also in the multivariate analysis, with *P* values less than 0.05. Stratified analyses were then performed according to person neoplasm cancer status, new tumor event after initial treatment, grade, respectively. First, all 309 HCC patients were stratified by person neoplasm cancer status into tumor free dataset (n = 135) and with tumor (n = 100) dataset. The six-mRNA signature could classify the tumor free dataset into a high-risk group (n = 84) and a low-risk group (n = 51) with significantly different survival. Similarly, the six-mRNA signature was also able to classify the with tumor dataset into a high-risk group (n = 51) and a low-risk group (n = 49) with significantly different survival (Figure 9A). Then all patients were further stratified by grade into an early dataset (grade I and grade II, n=182) and a late dataset (grade III and grade IV, n = 123). Similar prognostic power of the six-mRNA signature was significant in both the early dataset and late dataset. Patients in the early dataset were classified into a high-risk group (n = 91) with shorter survival and a low-risk group (n = 91) with longer survival. Similar results were observed in the late dataset (Figure 9C). Finally, all patients were stratified by the new tumor event after initial treatment into no dataset (n = 123) and yes dataset (n = 122). There was a significant difference in survival rate between high-risk group and low-risk group (Figure 9B). It is shown from that regression analysis of single variable and multivariable Cox and the result of stratified analysis that the prediction ability of six mRNA mark is independent from other clinical parameter and can predict the survival rate of HCC patients.

**Table 5:** Univariable analyses for each clinical feature.

**Clinical features**	**Hazard ratio**	**95% CI**	***p* Value**	**Hazard ratio**
Risk score	1.884	1.287-2.757	0.001	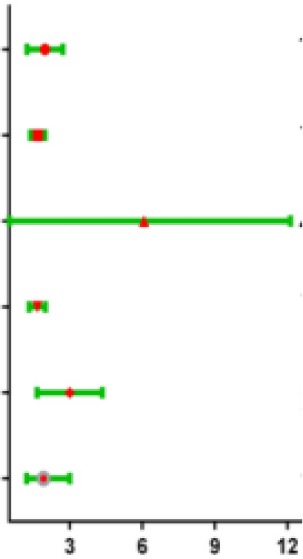
T	1.656	1.383-1.984	<0.001	
M	4.050	1.274-12.880	0.018	
Stage	1.636	1.337-2.003	<0.001	
Neoplasm cancer status	2.801	1.766-4.444	<0.001	
New tumor event after initial treatment	1.912	1.218-3.000	0.005	

Abbreviations: T, Tumor; N, Node(regional lymph node); M, Metastasis; 95% CI, 95% Confidence Interval

**Table 6:** Multivariable analyses for each clinical feature.

**Clinical features**	**Hazard ratio**	**95% CI**	***p* Value**	**Hazard ratio**
Risk score	1.884	1.287-2.757	<0.001	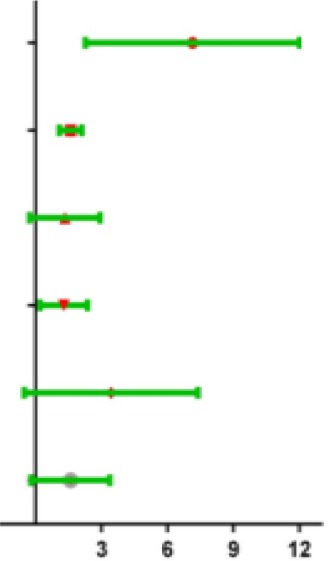
T	1.656	1.383-1.984	0.001	
M	4.050	1.274-12.880	0.617	
Stage	1.636	1.337-2.003	0.957	
Neoplasm cancer status	2.801	1.766-4.444	<0.001	
New tumor event after initial treatment	1.912	1.218-3.000	0.005	

Abbreviations: T, Tumor; N, Node(regional lymph node); M, Metastasis; 95% CI, 95%Confidence Interval

### Exploration of the differentially expressed genes between high-risk and low-risk patients related to the gene signature

To investigate the differentially expressed genes between high-risk and low-risk patients related to the gene signature, KEGG analysis was carried out on the risk score associated genes. By differential expression analysis, we extracted the differential expression data of 183 low-risk patients and 184 high-risk patients, and ordered them by *P* value. We selected the most 200 significant differentially expressed genes for subsequent analysis.

We found that these genes were enriched in terms including “cell cycle”, “protein digestion and absorption”, and“central carbon metabolism in cancer” (Supplementary Figure 1A). Meanwhile, using these genes with mRNA-signature, we performed PPI (protein-protein interaction) to find the hub gene through String (https://string-db.org/cgi/input.pl?sessionId= FB2xhX96kIxn&input_page_show_search=on) and cytoscape software. The result indicated that several genes for example, “CCNB1, CDK1, PLK1” (Supplementary Figure 1B) were the key genes we hoped to select, and “CENPA, STMN1, KIF2C, PTTG1” also played a crucial role in above pathways. Surprisingly, three genes of them that we identified before. It illustrated that “CENPA, STMN1, CDK1” are not only hub genes which have strong relationship with differentially expressed genes between high-risk and low-risk patients, but also the factors that influence the outcome of patients with HCC. All these results suggest that the novel gene signature reflects the HCC cellular functional characteristics related to the poor prognosis, thus predicts the survival of HCC patients.

### The protein of the six-genes were overexpressed in HCC tissues

**Figure 10:**
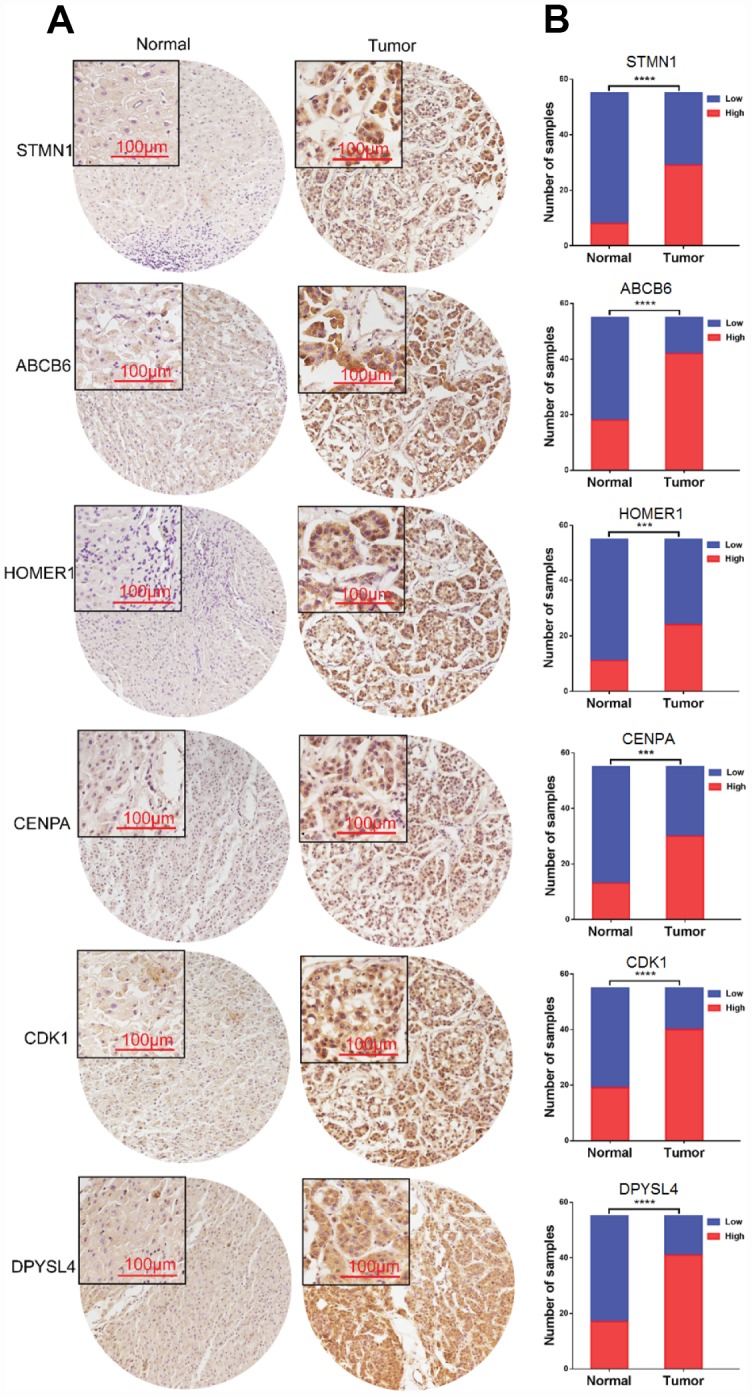
**Expression of six proteins in HCC tissues.** (**A**) Representative immunohistochemistry staining of the six proteins, (**B**) The sample number of high and low expression of each protein. (***p<0.001, ****p<0.0001).

**Figure 11:**
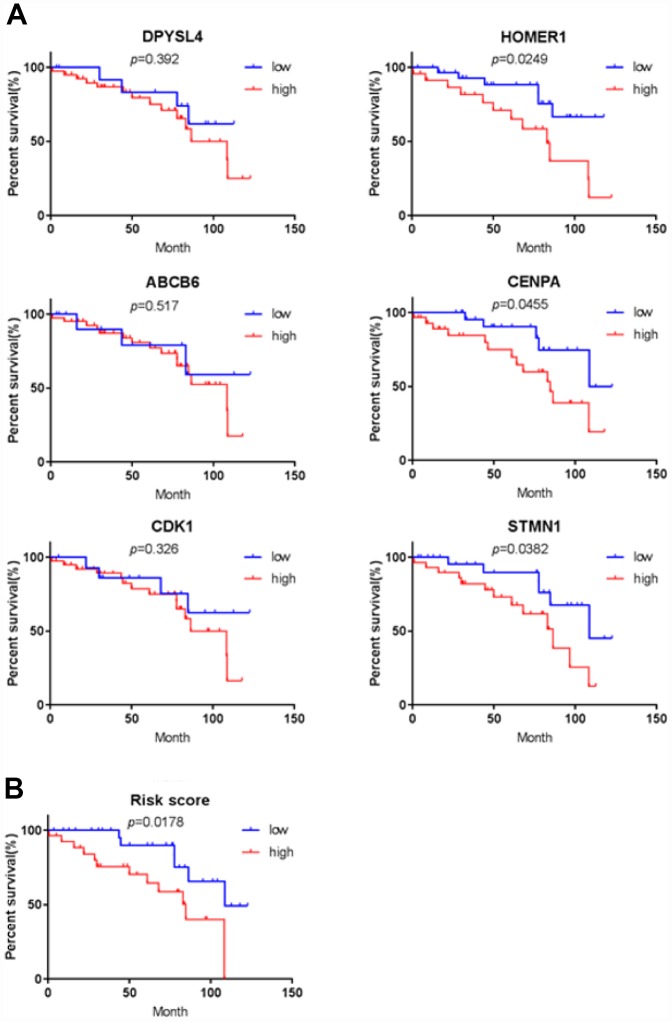
**Validation for prognostic value of six proteins and risk signature.** (**A**) K-M curves for DPYSL4, HOMER1, ABCB6, CENPA, CDK1 and STMN1, (**B**) K-M curves for risk score.

**Figure 12:**
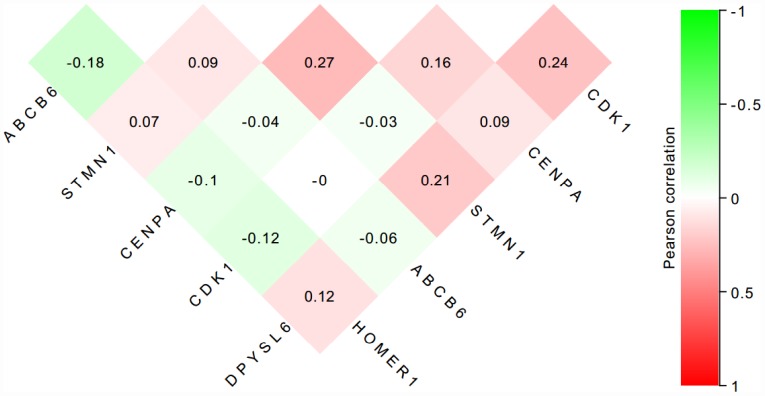
**The correlation of the six proteins in 55 HCC tissues.**

To further verify the protein expression level of the six-genes in HCC, immunohistochemistry was performed in TMA containing 55 paired HCC and adjacent tissue. Consistent with our previous findings, the six proteins were highly expressed in most HCC tissues compared with that in adjacent non-tumor liver tissues (Figure 10). As shown in Figure 10, high expression of STMN1 (29/55), ABCB6 (42/55), HOMER1 (24/55), CENPA (30/55), CDK1 (40/55) and DPYSL4 (41/55) were detected in a majority of HCC tissues, while high expression of STMN1 (8/55), ABCB6 (18/55), HOMER1 (11/55), CENPA (13/55), CDK1 (19/55) and DPYSL4 (17/55) were observed in a minority of adjacent non-tumor liver tissues. The potential association between protein expression and clinical features of HCC was analyzed. We found that high-expressed (STMN1, HOMER1, CENPA) patients had significant low overall survival rate compared with low-expressed group patients (Figure 11). These data indicate the survival time of high-risk group was significantly shorter than that of low-risk group, indicating that high-risk group had poor prognosis. We analyzed the correlation of the six proteins in HCC. The results showed that the absolute value of R values was less than 0.3, suggesting that these six proteins are independent of each other (Figure 12).

## DISCUSSION

In recent years, research on energy metabolism has been aroused people’s attention, furthermore, human malignancy and energy metabolism are inextricably linked. The metabolism of malignant tumors is characterized by the Warburg effect, in which a metabolic shift from oxidative phosphorylation in the mitochondria towards glycolysis occurs in tumor cells [9]. Although aerobic glycolysis is less efficient for ATP production than oxidative phosphorylation, aerobic glycolysis is essential for tumor growth and survival [10], by stimulating carbon fluxes to biosynthetic pathways. Increase NADPH synthesis and oxidative defense to meet cell proliferation needs [11]. In the case of cancer and energy metabolism, we have thought of biomarker, although there are many studies on cancer and glycolysis, the research that involved biomarkers of cancer related to glycolysis is limited. In our research this time, we tried to identify a glycolysis-related biomarkers for patients with HCC.

A biomarker is defined as any substance, structure or process that can be measured in the body and influence or predict the incidence of outcome or disease [12]. Recent studies demonstrated that clinicopathological features such as age, sex, tumor death, margin status, and metastatic diagnosis are insufficient to accurately predict patient prognosis [13]. The ideal biomarker is readily available, reliably measurable, cost-effective, minimally invasive and highly accurate [14]. Therefore, in the cancer, more and more mRNAs might be useful as molecular markers of the patients’ prognosis, indicating their important clinical significance should be explored [15]. For example, elevated GalNAc-T mRNA expression in melanoma cells appears to be a biomarker for aggressive melanoma [16], Han et al. confirmed that the MAGE family member A9 had significantly higher expression in laryngeal squamous cellcarcinoma and could be used as an independent prognostic factor in patients with laryngeal squamous cell cancer [17]. The expression of HERV-K in the PBMC has to do with the diagnosis of prostate cancer, particularly in elder men and smokers [18]. However, these biomarkers still exist to be insufficient to predict the survival of patients independently. In particular, because the expression of a single gene can be interfered by many factors through various ways, it is difficult to provide powerful functional predictive effects for such information. For this reason, here we use a statistical model to construct a gene signature containing several related genes, combined with the prediction effect of each component gene to improve prediction efficiency. This model is widely used and is superior to single biomarkers in predicting disease prognosis [19–21]. In our study, in order to confirm that the gene signature is more significant than the single gene biomarker, we verified it with the KM analysis and the ROC curve in HCC, and the results showed that our hypothesis is correct. When the six genes act as a single biomarker respectively, their diagnostic significance was not better than the six-mRNA signature (Figure 7). And for more in-depth research, the six genes and the mechanisms of glycolysis and HCC need further study.

Excluding in HCC, the above-mentioned methods (KM analysis and the ROC curve) combined with univariate and multivariate analysis were also performed in some other common cancers including KIRC, COAD, BRAC, OV to develop a glycolysis-related risk signature as a prognostic biomarker. And only HCC showed the strong relationship with glycolysis. In this regard, we conducted an in-depth discussion. When the results of GO enrichment analysis for target genes related with glycolysis were shown in HCC, we found that the most relevant enriched CC (Cellular Component) terms were associated with the golgi apparatus (Figure 3). The Golgi apparatus is a major glycosylation site of the cell and plays an essential role in the secretory pathway [22]. And it is the central organelle along the eukaryotic secretory and endocytic pathway [23]. Thus, the golgi apparatus is the most in cells that require a large amount of synthetic protein. In other words, cells with a secretory effect have more golgi bodies, for example, glandular cells (endocrine glands, digestive glands, etc.), as well as organs with high metabolic rates such as the liver. As the largest digestive gland in the human body, the liver can also secrete some immune globulins and some hormones. Considering that all of the above results are based on data analysis, we used immunohistochemical method to detect the protein level of these genes in HCC tissues. The results of immunohistochemistry were consistent with those of our data analysis, which confirmed that the expression level of these proteins in HCC tissues was significantly higher than that in adjacent tissues. In summary, when we develop a glycolysis-related risk signature as a prognostic biomarker, the result that only HCC showed the strong relationship with glycolysis is reasonable. Our findings provide novel insight to the relationship between glycolysis and HCC and we laid a solid foundation for future research.

In conclusions, we first identified and validated a six-gene risk signature related to glycolysis that can predict the outcome of patients with HCC, where higher risk scores indicate unfavorable survival. And we also provide novel insight into the relationship between glycolysis and HCC. This signature could be a promising prognostic targets in clinical practice, it is useful for verifying patients with HCC with poor prognoses. These results might offer a new view for the research of HCC and individual treatment.

## MATERIALS AND METHODS

### Patient clinical parameter and the genome expression data

Whole genome expression profiles and clinical data sets of tumor patients were extracted from the Cancer Genome Atlas Database (TCGA, The Cancer Genome Atlas, https://cancergenome.nih.gov/). In this study, we first downloaded the clinical pathological parameters and genome-wide expression profiles of four common solid tumors from the TCGA database, including colon adenocarcinoma, renal clear cell carcinoma, HCC, and breast invasive carcinoma. A total of 309 patients with HCC and 50 normal specimens with clinical features matched for subsequent study were included.

### Functional enrichment analyses

GSEA (http://www.broadinstitute.org/gsea/index.jsp) was performed to explore whether identified sets of genes showed significant differences between two groups [24]. GSEA does not require the specification of a clear differential gene threshold. The algorithm provides researchers with an overall trend of the actual data to enable the researchers to examine the overall expression of several genes even without prior experience; thus, this approach improves the connection between the mathematical statistics of the expression of the data and the biological meaning [25]. The expression levels of all mRNAs in adjacent noncancerous tissue and tumor samples were analyzed. Normalized *p* values (*P*<0.05) were used to determine which gene set to further investigate. Functional enrichment analyses for those filtered genes were performed using the DAVID (The Database for Annotation, Visualization and Integrated Discovery) Bioinformatics Tool (version 6.7) [26]. Significant enrichment results were visualized using EasyChart (http://www.ehbio.com/ImageGP/index.php/Home/Index/index.html).

### Prognostic analysis

The relationship between the expression level of each mRNAs and the patient OS was calculated using a univariate Cox model. In univariate variable Cox analysis, mRNAs with p values less than 0.05 were considered statistically significant. After that, the multi-variable Cox analysis was used to evaluate the weight of mRNAs as the independent predictor of survival. These analyses were conducted using the R package of survival.

### Statistical analysis

The filtered mRNAs were classified into risk (HR > 1) and protective (0 < HR < 1) types. Subsequently, a prognostic risk score formula was established based on a linear combination of the expression levels weighted with the regression coefficients derived from the multivariate Cox regression analysis.

Risk score = expression of gene 1×β_1_ + expression of gene 2×β_2_ +......+ expression of gene n×β_n_. We classified 309 patients into high-risk and low-risk subgroups using the median risk score as the cutoff. Kaplan-Meier curves and the log-rank method were used to validate the prognostic significance of the risk score in stratified analysis. The Student's t test was performed to examine the differential expression of optimal genes in adjacent normal tissue and GC tissues. All of the statistical analyses were performed using SPSS 16.0 and GraphPad Prism 7.0 software. We also researched the genetic alterations of prognosis-related genes in HCC by cBioPortal web software (http://www.cbioportal.org/). Chi-square test was used to demonstrate the relationship between risk score and clinical parameters. Scikit-learn package of Python was used to do the cross validation.

### Tissue microarray construction and immunohistochemistry

After reviewing the hematoxylin and eosin-stained slides, 55 paired representative paraffin blocks (2007-2017) of HCC and adjacent tissue samples were selected. Tissue cores were extracted from each donor block using a 1.5 mm diameter puncture needle, and arrayed into a new paraffin recipient block with a maximum of 60 cores. Sections were obtained from re-prepared blocks, mounted on poly-L-lysine-coated glass slides, and used for immunohistochemical staining. Sections were deparaffinized and exposed to antigen retrieval at 121°C for 5 min using an autoclave (pH 7.8 Tris-EDTA-citrate buffer). Sections were then incubated with primary antibodies against DPYSL4, HOMER1, ABCB6, CENPA, CDK1 or STMN1 (Sangon Biotech, China) at 37°C for 60 min, followed by incubation with biotinylated secondary antibodies at 37°C for 30 min. The sections were then incubated with horseradish peroxidasecoupled streptavidin for additional 30 min (LSAB kit; Dako, Glostrup, Denmark), and stained with DAB. Sections were then dehydrated.

### Evaluation of immunohistochemistry

The immunostaining was evaluated under the light microscope (magnification, ×200; select three fields/ view) by two pathologists blinded to the experimental conditions. The intensity of immunoreactivity was scored as follows: zero for no staining, one for weak staining, two for moderate staining, and three for strong staining. The proportion of positive tumor cells was as follows: zero (no positive cells), one (<25% positive cells), two (26-50% positive cells), three (51-75% positive cells), four (>75% positive cells). The score is obtained by calculating the product of intensity of immunoreactivity and proportion of positive tumor cells. Score >= six represents high expression, otherwise it is low expression.

## Supplementary Material

Supplementary Figure 1

Supplementary Table 1
