# Supplementary material for: Glycolysis gene expression profilings screen for prognostic risk signature of hepatocellular carcinoma
Source: Aging (Albany NY). 2019 Dec 2;11(23):10861–82. doi: 10.18632/aging.102489 (PMC6932884; doi:10.18632/aging.102489)
Supplement: Supplementary Figure 1 [file aging-11-102489-s002..pdf]

## SUPPLEMENTARY FIGURE

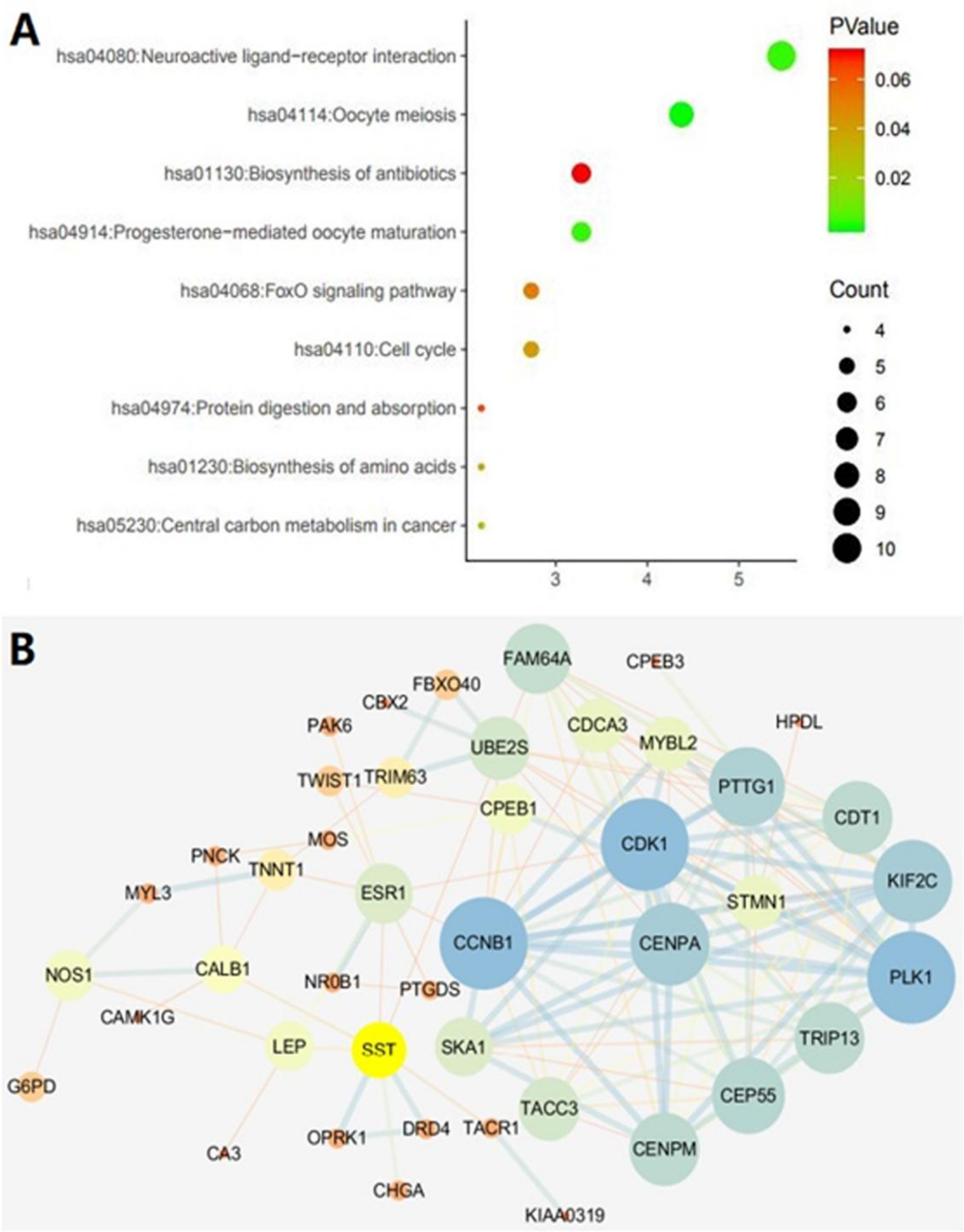


**Supplementary Figure 1. The differentially expressed genes between high-risk and low-risk patients related to the gene signature.** (**A**) Enrichment plots of differentially expressed genes, (**B**) Networks regulated by risk score-associated genes in HCC constructed by Cytoscape. (Low degree to bright colors for map nodes color, low degree to small sizes for map nodes size, Low combined-score to small sizes for map edge size.)
